# Cognitive Dysfunction in Type 2 Diabetes Is Not a One-Way Process: Evidence From a Longitudinal Brain Connectivity Study

**DOI:** 10.3389/fendo.2022.874538

**Published:** 2022-04-28

**Authors:** Fang Fang, Yu-Jia Gong, Qian Luo, Ren-Bin Ge, Mei Kang, Ming-Ming Ma, Lei Zhang, Di Mu, Da-Zhi Yin, Yu-Fan Wang

**Affiliations:** ^1^ Department of Endocrinology and Metabolism, Shanghai General Hospital, Shanghai Jiao Tong University, Shanghai, China; ^2^ Department of Radiology, Shanghai General Hospital, Shanghai Jiao Tong University, Shanghai, China; ^3^ Clinical Research Center, Shanghai General Hospital, Shanghai Jiao Tong University, Shanghai, China; ^4^ Shanghai Key Laboratory of Ocular Fundus Diseases, Shanghai Engineering Center for Visual Science and Photomedicine, Shanghai General Hospital, Shanghai Jiao Tong University, Shanghai, China; ^5^ Department of Anesthesiology, Shanghai General Hospital, Shanghai Jiao Tong University, Shanghai, China; ^6^ Shanghai Key Laboratory of Brain Functional Genomics (Ministry of Education), School of Psychology and Cognitive Science, East China Normal University, Shanghai, China; ^7^ Shanghai Changning Mental Health Center, Shanghai, China

**Keywords:** cognitive reversal, hippocampus, functional connectivity, magnetic resonance imaging, type 2 diabetes, young adult

## Abstract

**Background:**

Cognitive dysfunction is an important comorbidity of diabetes characterized by brain functional hypo-connectivity. However, our recent study demonstrated an adaptive hyper-connectivity in young type 2 diabetes with cognitive decrements. This longitudinal study aimed to further explore the changes in functional connectivity and cognitive outcomes after regular glycemic control.

**Methods:**

At 18 months after recruitment, participants underwent a second cognitive assessment and magnetic resonance imaging. Three enhanced functional connectivities previously identified at baseline were followed up. Linear mixed-effects models were performed to compare the longitudinal changes of cognition and functional connectivity in patients with type 2 diabetes and non-diabetic controls. A linear regression model was used to investigate the association between changes in functional connectivity and changes in cognitive performance.

**Results:**

Improvements in multiple cognitive domains were observed in diabetes; however, the enhanced functional connectivity at baseline decreased significantly. Moreover, the decrease in hippocampal connectivity was correlated with an increase in the accuracy of Stroop task and the decrease in posterior cingulate cortex connectivity was correlated with an increase in Montreal Cognitive Assessment in diabetes.

**Conclusion:**

This study suggests diabetes-related cognitive dysfunction is not a one-way process and the early-stage enhancement of brain connectivity was a potential “window period” for cognitive reversal.

## Introduction

Patients with type 2 diabetes are at an increased risk of cognitive dysfunction, ranging from subtle cognitive deficits to major neurocognitive disorders (1). Severe cognitive dysfunction mainly occurs in patients older than 65 years, whereas subtle cognitive deficits have been observed in diabetes of all age groups (2). Despite being within the normal cognitive range and not affecting daily life, the presence of slight cognitive deficits might be additive to the accelerated cognitive decline and might reduce the symptomatic threshold of dementia (3). Given the growing prevalence of type 2 diabetes in young individuals worldwide, efforts to elucidate the changes in brain neurophysiology in young patients have become essential and urgent.

Noninvasive multimodal magnetic resonance imaging (MRI) (e.g., structural and functional) techniques provide potential biomarkers for cognitive dysfunction (4). Previous neuroimaging studies have consistently demonstrated modest brain atrophy (5) and reduced functional connectivity among brain regions (6) in middle-aged to elderly patients with type 2 diabetes. For example, based on resting-state functional MRI (fMRI), the functional connectivity between seed regions (e.g., bilateral hippocampus (7, 8) and posterior cingulate cortex [PCC] (9, 10)) and multiple regions in the default mode network was weaker in diabetes than in controls and the decreased connectivity was correlated with cognitive decline.

In contrast, in our recent study, young adults with type 2 diabetes (age: <40 years) at an early stage of disease progression (no detectable microvascular complications) had an enhanced resting-state functional connectivity compared with non-diabetes controls, which is related to cognitive decrements and occurs before brain morphometric change (11). These findings suggest a compensation of brain function to counteract the insidious cognitive decline during the early stage of type 2 diabetes. Moreover, this increase in intrinsic functional connectivity has been found in young type 1 diabetic children (12) and in type 1 diabetic adults without microvascular complications (13). Overall, the emerging neuroimaging studies suggest nonlinear changes in brain connectivity at different stages of diabetes progression. However, the evolution of brain hyperconnectivity in the early stages of diabetes and its effects on neuropsychological performance under glycemic control have not been demonstrated.

In the current longitudinal study, the three enhanced functional connectivities previously identified at baseline (i.e., connectivity of the left hippocampus with the left inferior frontal gyrus [IFG] and left inferior parietal lobule [IPL] and connectivity of the posterior cingulate cortex [PCC] with the left IPL) were further investigated both in young adults with type 2 diabetes and controls. In addition, voxel-based morphometric (VBM) analysis was performed to investigate the change in brain volume. We hypothesized that the enhancement of functional connectivity was temporary, which would disappear and contribute to the change of cognitive function.

## Materials and Methods

### Participants and Recruitment

This was an extension of our previous cross-sectional study (11). The recruited patients with type 2 diabetes (age: < 40 years) and non-diabetic controls were followed up. Patients had no peripheral microvascular complications such as diabetic retinopathy, diabetic nephropathy, and diabetic peripheral neuropathy at recruitment. Participants with a history of diabetic ketosis or ketoacidosis, hypoglycemia within 48 hours, clinical evidence of cardiovascular or cerebrovascular diseases, a history of alcohol consumption, thyroid dysfunction, anemia, and any MRI contraindications were excluded. After recruitment, patients were provided diabetes education and individualized treatment. All licensed antidiabetic medications were permitted. No target hemoglobin A1c was predetermined. The follow-up visits targeted at 18 months after recruitment. Of the 67 participants (35 patients with type 2 diabetes and 32 controls) recruited at baseline, 50 (74.6%) participants (26 patients with type 2 diabetes and 24 controls) consented to participate in the extensional study. Participants were given separate informed consents for this longitudinal study. The study protocol was reviewed and approved by Shanghai General Hospital Ethics Committee, approval number 2019SQ082.

### Neuropsychological and Clinical Assessments

Cognitive function was assessed at both time-points using the same methodology (11) and by the same physician. Briefly, the cognitive battery included tests for global cognitive function (Montreal Cognitive Assessment [MoCA]), executive function (the accuracy and reaction time of Stroop Color Word Test - part C), memory function (Rey Auditory Verbal Learning Test [RAVLT]), and language function (Verbal Fluency Test [VFT] and Boston Naming Test [BNT]).

Age, sex, education level, and follow-up interval (i.e., the time from baseline to follow-up MRI scan) were recorded for statistical correction. The levels of hemoglobin A1c (HbA1c), fasting plasma glucose, and fasting serum C-peptide were assessed to determine the glycemic control at the two time points. Homeostasis model assessment (HOMA) (14) was used to assess β-cell function (HOMA-%β) and insulin resistance (HOMA-IR). Antidiabetic drugs used by patients with type 2 diabetes at recruitment and follow-up visits were recorded. The laboratory data were collected a day before the MRI scan.

Microvascular complications were assessed in patients with diabetes at the two time points. A dilated fundus examination using a 90-diopter pan-fundus lens was performed by an ophthalmologist. Diabetic retinopathy was defined as the presence of any of the following lesions: retinal microaneurysms, hemorrhages, hard exudates, soft exudates, neovascularization, or evidence (or history) of laser photocoagulation. The urinary albumin-to-creatinine ratio (UACR) was measured in first-void clean-catch urine samples collected on two consecutive days. The average UACR for two consecutive days was used for the analysis.

### MRI Data Acquisition and Preprocessing

The baseline and follow-up MRI were performed using a 3-Tesla MRI scanner (Ingenia, Philips Healthcare, Best, NL). fMRI data were acquired using a gradient-echo-planar imaging sequence with the same parameters and instructions (e.g., eyes closed): repetition time (TR), 2000 ms; echo time (TE), 30 ms; field of view, 224 × 224 mm^2^; flip angle, 90°; slices, 33; slice thickness, 3.5 mm; slice spacing, 0.7 mm; matrix, 64 × 62; volumes, 240; and acquisition time, 8 min. High-resolution, T1-weighted images were obtained using a magnetization-prepared rapid gradient-echo sequence (TR = 7.0 ms, TE = 3.2 ms, flip angle = 7°, inversion time = 1100 ms, and voxel size = 1 × 1 × 1 mm^3^).

The fMRI data were preprocessed using Data Processing Assistant for Resting-State fMRI software (15) and the following parameters, as in our previous study (11): 1) slice timing; 2) spatial realignment to correct for head motion; 3) spatial normalization of images to the Montreal Neurological Institute (MNI) space using a unified segmentation algorithm (16) and resampling to 3-mm isotropic voxels; 4) spatial smoothing with a 6-mm full-width-half-maximum (FWHM) Gaussian kernel; and 5) linear detrending, bandpass filtering (0.01–0.08 Hz), and regressing out several covariates such as six head-motion parameters and white matter (WM) and cerebrospinal fluid (CSF) signals.

### Clinical and Neuropsychological Analyses

Subjects with both baseline and follow-up data were enrolled for the statistical analysis. The between-group differences at each time point were analyzed using a two-tailed two-sample t-test, Mann–Whitney U test, or chi-square test. Neuropsychological scores were normalized by logarithmic transformation. Thereafter, linear mixed-effects models were performed to compare the longitudinal changes of neuropsychological scores in patients with diabetes and non-diabetic controls between the two time-points, with adjustment for sex, age at baseline, education level and follow-up interval.

### Resting-State Functional Connectivity Analysis

Functional connectivity analysis using the Resting-State fMRI Data Analysis Toolkit (REST, http://restfmri.net) was performed using bilateral hippocampus and PCC as the seed region of interest (ROI) in our previous study (11). The three enhanced functional connectivities which were founded in previous between-group analysis were further investigated. The value of each functional connectivity for each subject at both time points was extracted. We first performed between-group analysis at each time point using a two-tailed two-sample t-test. We then performed linear mixed-effects models to compare the longitudinal changes of brain functional connectivity in patients with diabetes and controls between the two time-points, adjusted for sex, age at baseline, education level and follow-up interval.

Finally, to determine the associations between the changes in the three functional connectivities and the changes in neuropsychological performance, we performed linear regression analyses adjusting sex, age at baseline, education level, and follow-up interval in each group. The associations between changes in the aforementioned connectivity and changes in hyperglycemia-related variables (i.e., HbA1c level, fasting plasma glucose level, fasting serum C-peptide level, HOMA-%β, and HOMA-IR) were also determined in patients with diabetes.

### VBM Analysis

We performed VBM analysis on T1-weighted images using the VBM8 toolbox in the SPM8 software package (http://www.fil.ion.ucl.ac.uk/spm/). Cerebral tissues from each participant were segmented into gray matter (GM), WM, and CSF. Images of each participant’s GM, WM, and CSF were nonlinearly registered using the DARTEL method and transformed into the MNI standard space. The warped GM images were modulated by Jacobian determinants to include the information of volume in these modulated images. Finally, the resultant maps were smoothed with a 6-mm FWHM Gaussian kernel. In addition, the GM, WM, CSF, and total brain volumes for each participant were obtained simultaneously.

At the voxel level, between-group differences in the GM volume were determined using random-effects two-sample t-tests with age, sex, educational level, and BMI as nuisance covariates. The threshold was set at a corrected *P* value of < 0.05, with multiple comparisons corrected using the AlphaSim program (http://afni.nimh.nih.gov/pub/dist/doc/manual/AlphaSim.pdf) determined by 1000 Monte Carlo simulations (i.e., single voxel *P* < 0.001, combining a minimum cluster size). In addition, we performed between-group analysis for the mean volumes of GM, WM, and CSF, as well as total brain volume.

## Results

### Demographic and Clinical Data

The mean follow-up intervals for patients and controls were 18.0 ± 5.5 and 18.0 ± 4.9 months respectively (*P* = 0.891). The characteristics of subjects who withdrew from the study are presented in **Supplemental Table 1**.

Compared with the controls, the patients with type 2 diabetes had higher body mass index (*P* = 0.017), HbA1c level (*P* < 0.001), fasting blood glucose (*P* = 0.011), HOMA-%β (*P* = 0.003), and lower HDL cholesterol level (*P* = 0.002) at the follow-up visit as expected. However, there were no differences in the level of HOMA-%β (*P* = 0.965), triglyceride (*P* = 0.083), and uric acid (*P* = 0.129) at the follow-up visit, and these variables were statistically different between groups at baseline. Notably, the number of patients who were taking antidiabetic medicines increased from 42.3% to 92.3% from baseline to follow-up. A large proportion of patients were using metformin (73.1%) and/or glucagon-like peptide 1 receptor agonists (42.3%) at the follow-up visit. The demographic and clinical data of the participants are shown in **Table 1**.

**Table 1:** Demographic and Clinical data at baseline and at the follow-up visit.

	Baseline			Follow-up		
	Controls N = 24	Diabetes N = 26	*P* values	Controls N = 24	Diabetes N = 26	*P* values
**Age, year**	34.1 ± 4.4	33.0 ± 5.5	0.444	35.4 ± 4.2	34.6 ± 5.6	0.574
**Male**	13 (54.2)	20 (76.9)	0.090	13 (54.2)	20 (76.9)	0.090
**Education, year**	14.2 ± 4.6	13.2 ± 2.7	0.324	14.2 ± 4.6	13.2 ± 2.7	0.324
**Body Mass Index, kg/m^2^**	23.8 ± 3.0	26.7 ± 3.7	0.004^*^	23.9 ± 3.0	26.1 ± 3.5	0.017^*^
**History of smoking**	10 (41.7)	10 (38.5)	0.817	10 (41.7)	11 (42.3)	0.963
**Presence of hypertension**	5 (20.8)	8 (30.8)	0.424	5 (20.8)	8 (30.8)	0.424
**Statin treatment**	0 (0)	5 (19.2)	0.051	0 (0)	7 (26.9)	0.010^*^
**Diabetes treatment**	–	11 (42.3)	–	–	24 (92.3)	–
**Metformin**	–	9 (34.6)	–	–	19 (73.1)	–
**Sulfonylureas**	–	3 (11.5)	–	–	0 (0)	–
**Glinides**	–	2 (7.7)	–	–	1 (3.8)	–
**Thiazolidinediones**	–	0 (0)	–	–	1 (3.8)	–
**Acarbose**	–	2 (7.7)	–	–	8 (30.8)	–
**DPP-4i**	–	2 (7.7)	–	–	7 (26.9)	–
**SGLT-2i**	–	(0)	–	–	2 (7.7)	–
**GLP-1A**	–	(0)	–	–	11 (42.3)	–
**Insulin**	–	3 (11.5)	–	–	4 (15.4)	–
**HbA1c, %**	5.5 ± 0.3	10.0 ± 2.2	< 0.001^*^	5.2 ± 0.2	6.5 ± 1.2	< 0.001^*^
**Fasting plasma glucose, mmol/L**	4.74 ± 0.55	8.45 ± 3.55	< 0.001^*^	5.16 ± 0.25	7.14 ± 3.07	0.011^*^
**Fasting serum C-peptide, pmol/L**	507.0 ± 200.7	578.3 ± 306.2	0.372	449. 0 ± 190.2	673.1 ± 326.8	0.015^*^
**HOMA-%β**	116.6 ± 41.6	53.4 ± 30.7	< 0.001^*^	85.8 ± 21.1	85.1 ± 54.0	0.965
**HOMA-IR**	1.09 ± 0.43	1.58 ± 0.98	0.046^*^	0.94 ± 0.39	1.62 ± 0.77	0.003^*^
**Total Cholesterol, mmol/L**	4.92 ± 1.14	4.99 ± 0.94	0.805	4.75 ± 0.97	4.80 ± 0.75	0.837
**Triglyceride, mmol/L**	1.31 ± 0.79	2.74 ± 1.50	< 0.001^*^	1.56 ± 1.39	2.60 ± 2.13	0.083
**HDL cholesterol, mmol/L**	1.32 ± 0.34	0.90 ± 0.23	< 0.001^*^	1.25 ± 0.30	0.99 ± 0.21	0.002^*^
**LDL cholesterol, mmol/L**	3.14 ± 0.89	3.08 ± 0.77	0.809	2.59 ± 0.76	2.57 ± 0.78	0.944
**Uric acid, μmol/L**	296.0 ± 80.2	372.7 ± 134.0	0.023^*^	334.8 ± 92.0	391.7 ± 130.4	0.129
**Urine ACR, mg/g**	9.38 (4.5, 21.7)	14.0 (2.1, 29.9)	0.058	9.08 (5.9, 28.0)	6.25 (2.5, 307)	0.419
**Diabetic retinopathy**	–	0 (0)	–	–	3 (1.2)	–
**Diabetes duration, year**	–	1.5 (0, 10)	–	–	2.8 (1.1, 11.3)	–

Data are represented as mean ± SD, n (%), or median (range). *P <0.05. DPP-4i, Dipeptidyl peptidase 4 inhibitors; SGLT-2i, Sodium glucose cotransporter 2 inhibitors; GLP-1A, Glucagon-like peptide 1 receptor agonists; HOMA-%β, homeostasis model assessment of β-cell function; HOMA-IR, homeostasis model assessment of insulin resistance; HDL, high-density lipoprotein; LDL, low-density lipoprotein; and ACR, albumin-to-creatinine ratio.

### Neuropsychological Tests

**Figure 1:**
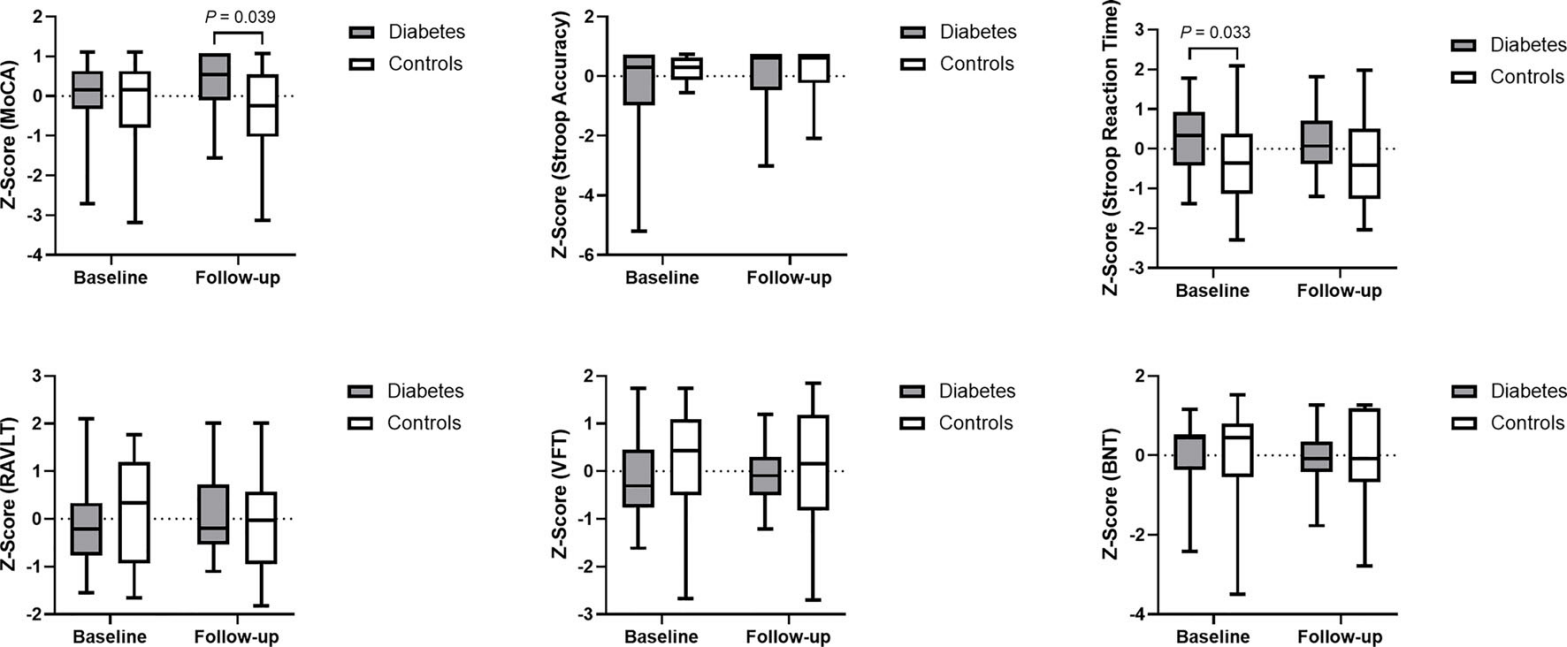
Data distribution and between-group comparison of cognitive performance. Z-score was used for standardization of data. Higher cognitive scores and lower Stroop Reaction Time indicate better cognitive function. MoCA, Montreal Cognitive Assessment; Stroop Accuracy, the accuracy of Stroop Color Word Test - part C; Stroop Reaction Time, the reaction time of Stroop Color Word Test - part C; RAVLT, Rey Auditory Verbal Learning Test; VFT, Verbal Fluency Test; and BNT, Boston Naming Test.

The between-group differences in the neuropsychological tests at baseline are consistent with our former study, which revealed longer Stroop Reaction Time in patients with type 2 diabetes than in controls (*P* = 0.033 in the current sample), indicating poorer executive function in the patients group. At the follow-up visit, interestingly, patients with type 2 diabetes had a higher MoCA than the controls (*P* = 0.039), which indicated better global cognitive function in the patients group. No significant differences were noted in any other neuropsychological performances between the two groups at the follow-up visit (**Figure 1**).

The linear mixed-effects model demonstrated that the longitudinal trajectories of log-transformed MoCA and RAVLT differed significantly between the two groups. Compared with controls, patients with diabetes had a 1.5% greater incremental rate of MoCA score (β = 0.015, 95% CI: 0.00013 – 0.030) and a 5% greater incremental rate of RAVLT (β = 0.050, 95% CI: 0.0002 – 0.100) on a logarithmic scale from baseline to follow-up visit. Furthermore, significant increase of log-transformed Stroop Accuracy ((β = -0.009, 95% CI: -0.016 – -0.002) and decrease of Stroop Reaction Time ((β = 0.030, 95% CI: 0.010 – 0.049)) were observed among patients with diabetes, which indicated that patients had better executive function at follow-up compared to that at baseline.

### Functional Connectivity Measures

**Figure 2:**
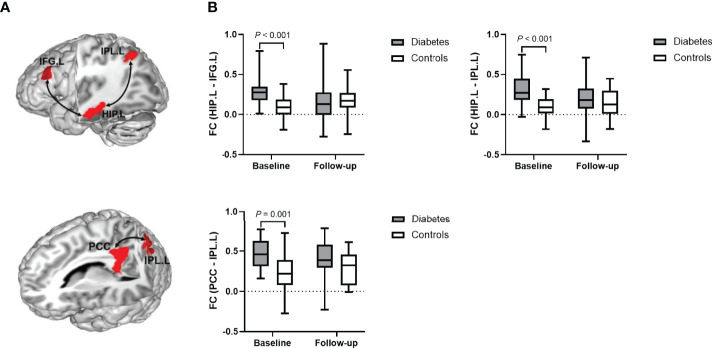
**(A)** A representative model of the enhanced functional connectivity at baseline. **(B)** Data distribution and between-group comparisons of functional connectivity. HIP.L, left hippocampus; IFG.L, left inferior frontal gyrus; IPL.L, left inferior parietal lobule; PCC, posterior cingulate cortex; and FC, functional connectivity.

Baseline between-group analyses revealed that the three functional connectivities were significantly higher in patients with type 2 diabetes than in the controls (*P* < 0.001 for the connectivity of the left hippocampus with the left IFG and the left IPL; *P* = 0.001 for the connectivity of the PCC with the left IPL), which were also consistent with our former study. At the follow-up visit, however, the between-group differences disappeared (**Figure 2**).

Moreover, the linear mixed-effects model demonstrated the longitudinal changes of the connectivity of the left hippocampus with the left IFG and the left IPL differed significantly between diabetic group and controls. Compared with controls, patients with diabetes had a 20.7% greater decremental rate in the connectivity of the left hippocampus with the left IFG (β = -0.207, 95% CI: -0.334 – -0.080) and a 16.0% greater decremental rate in the connectivity of the left hippocampus with the left IPL (β = -0.160, 95% CI: -0.271 – -0.048) from baseline to follow-up visit (**Table 2**).

**Table 2:** Linear mixed-effects models for the changes of cognition and functional connectivity in patients with diabetes and controls from baseline to follow-up visit.

	β	95% CI	*P* values
**Cognition (Log-transformed)**			
** MoCA**			
Group	-0.022	(-0.034, -0.009)	0.001^*^
Visit	-0.012	(-0.022, -0.002)	0.026^*^
Group * Visit	0.015	(0.0001, 0.030)	0.048^*^
** Stroop Accuracy**			
Group	0.002	(-0.003, 0.007)	0.385
Visit	-0.009	(-0.016, -0.002)	0.011^*^
Group * Visit **^†^**	0.011	(-0.003, 0.024)	0.111
** Stroop Reaction Time**			
Group	-0.037	(-0.082, 0.007)	0.100
Visit	0.030	(0.010, 0.049)	0.004^*^
Group * Visit **^†^**	-0.009	(-0.048, 0.030)	0.636
** RAVLT**			
Group	-0.045	(-0.095, 0.005)	0.078
Visit	-0.053	(-0.087, -0.018)	0.004^*^
Group * Visit	0.050	(0.0002,0.100)	0.049^*^
** VFT**			
Group	0.005	(-0.040, 0.050)	0.818
Visit	-0.007	(-0.024, 0.011)	0.452
Group * Visit **^†^**	0.017	(-0.017, 0.052)	0.324
** BNT**			
Group	-0.0007	(-0.025, 0.024)	0.956
Visit	-0.008	(-0.017, 0.0009)	0.078
Group * Visit **^†^**	0.005	(-0.013, 0.023)	0.537
**Functional Connectivity**			
** HIP.L-IFG.L**			
Group	0.026	(-0.101, 0.152)	0.684
Visit	0.146	(0.058, 0.234)	0.002^*^
Group * Visit	-0.207	(-0.334, -0.080)	0.002^*^
** HIP.L-IPL.L**			
Group	-0.041	(-0.160, 0.079)	0.495
Visit	0.100	(0.023, 0.178)	0.012^*^
Group * Visit	-0.160	(-0.271, -0.048)	0.006^*^
** PCC-IPL.L**			
Group	-0.129	(-0.259, 0.002)	0.053
Visit	-0.050	(-0.041, 0.141)	0.276
Group * Visit **^†^**	-0.122	(-0.253, 0.010)	0.069

Model adjusted for sex, age at baseline, education level and follow-up interval. **^†^** Interaction was not adopted in the final model since there was no statistical difference of the interactive effect. ^*^P <0.05.

β coefficient for “Group” represents the difference of the variable (i.e., MoCA, Stroop Accuracy, Stroop Reaction Time, RAVLT, VFT, BNT, FC [HIP.L-IFG.L], FC [HIP.L-IPL.L], or FC [PCC-IPL.L]) levels between patients with diabetes and non-diabetic controls at the follow-up visit.

β coefficient for “Visit” represents the longitudinal change of the variable (i.e., MoCA, Stroop Accuracy, Stroop Reaction Time, RAVLT, VFT, BNT, FC [HIP.L-IFG.L], FC [HIP.L-IPL.L], or FC [PCC-IPL.L] levels) levels from baseline to follow-up visit among patients with diabetes.

β coefficient for “Group * Visit” represents the difference in longitudinal change of the variable (i.e., MoCA, Stroop Accuracy, Stroop Reaction Time, RAVLT, VFT, BNT, FC [HIP.L-IFG.L], FC [HIP.L-IPL.L], or FC [PCC-IPL.L] levels) levels from baseline to follow-up visit between patients with diabetes and non-diabetic controls.

MoCA, Montreal Cognitive Assessment; Stroop Accuracy, the accuracy of Stroop Color Word Test – part C; Stroop Reaction Time, the reaction time of Stroop Color Word Test – part C; RAVLT, Rey auditory verbal learning test; VFT, Verbal fluency test; BNT, Boston naming test; FC (HIP.L-IFG.L), the functional connectivity of the left hippocampus with the left inferior frontal gyrus; FC (HIP.L-IPL.L), the functional connectivity of the left hippocampus with the left inferior parietal lobule; FC (PCC-IPL.L), the functional connectivity of the posterior cingulated cortex with the left inferior parietal lobule.

Considering that the antidiabetic drugs might have a direct effect on brain functional connectivity (7), we further divided the patients into two subgroups according to whether they took antidiabetic drugs at baseline (Medicated subgroup, *N* = 11; non-Medicated subgroup, *N* = 15) to obtain a comprehensive understanding of the brain neurophysiology in young patients with type 2 diabetes. Between-subgroup analyze demonstrated that the functional connectivity between PCC and left IPL was lower in medicated subgroup compared with that in non-medicated subgroup at baseline. No between-subgroup differences were observed in cognitive performance at baseline, the changes of cognitive performance and the changes of functional connectivity from baseline to the follow-up visit (**Supplemental Table 2**). The decreased intrinsic functional connectivity in medicated subgroup at baseline provided some clues to the further research on the effect of antidiabetic drugs on brain function.

### Brain Volume Measures

No significant between-group differences were observed in brain morphometric analyses (both voxel-based and global brain volume analyses) at both time-points. (**Supplemental Table 3**).

### Regression Analysis

**Figure 3:**
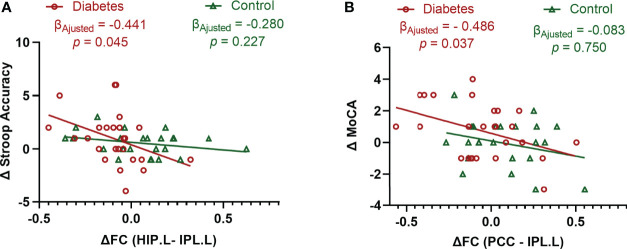
Associations between the changes in cognitive performance and the changes in functional connectivity. **(A)** Significant association between the changes in Stroop Accuracy and the changes in the functional connectivity between the left hippocampus and left inferior parietal lobule in type 2 diabetic patients after adjusting for age, sex, education level, and follow-up interval. **(B)** Significant association between the changes in MoCA and the changes in the functional connectivity between the posterior cingulate cortex and left inferior parietal lobule in type 2 diabetic patients after adjusting for age, sex, education level, and follow-up interval. Δ is the value obtained after subtracting the baseline value from the follow-up value. Data exceeding 2.5 Standard Deviation were removed from the analyses (Analyses with original data are in **Supplemental Figure 1**). Stroop Accuracy, the accuracy of Stroop Color Word Test - part C; MoCA, Montreal Cognitive Assessment; HIP.L, left hippocampus; IPL.L, left inferior parietal lobule; PCC, posterior cingulate cortex; and FC, functional connectivity.

In patients with type 2 diabetes, the change in Stroop Accuracy was negatively correlated with the change in functional connectivity between the left hippocampus and the left IPL after adjusting for age at baseline, sex, education level, and follow-up interval (β = -0.441, *P* = 0.045). This indicated that the greater the decline in the hippocampal connectivity, the greater the improvement of executive function. Moreover, the change in MoCA was negatively correlated with the change in functional connectivity between the PCC and left IPL after adjusting for the aforementioned potential confounders (β = −0.486, *P* = 0.037), indicating that the greater the decline in PCC connectivity, the greater the improvement of global cognition. In contrast, no association was observed between the changes in functional connectivity and the changes in cognitive performance in the controls (**Figure 3**).

The associations between the changes in the three functional connectivities and the changes in the HbA1c level as well as other hyperglycemia-related variables (fasting plasma glucose level, fasting serum C-peptide level, HOMA-%β, and HOMA-IR) were also investigated in patients with type 2 diabetes. However, no significant associations were found after adjusting for potential confounders (**Supplemental Table 4**).

## Discussion

Diabetes-associated cognitive dysfunction progresses extremely insidiously and is almost irreversible once the pathology is present in the brain (17). However, the current longitudinal study demonstrated an improvement in cognition in young type 2 diabetic patients with early stage disease after a mean follow-up of 18.0 months. Increased cognition abated baseline cognitive decrements of the diabetic patients, indicating a cognitive reversal.

Meanwhile, the enhanced functional connectivity at baseline was significantly decreased in the patients with type 2 diabetes, resulting in a level similar to that of the controls at the follow-up visit. Intriguingly, the decline in the connectivity of the left hippocampus with the left IPL was related to the increase in executive function, whereas the decrease in the connectivity of the PCC with the left IPL was related to the increase in MoCA. IPL is a major hub (critically important for information integration) of the frontoparietal control system (18) which is involved in processing a diverse range of higher cognitive functions (19). It suggests that the baseline functional hyperconnectivity could be normalized in response to improved cognition. Therefore, the early brain hyperconnectivity might serve as a biomarker for an important preclinical stage, or so-called “window period” for cognitive reversal. During the “window period,” pathological changes in the brain are not accumulated. However, brain function is disturbed by risk factors such as hyperglycemia, which can be detected by brain connectivity.

Practice effects of repeated cognitive testing, which are often observed in cognitively healthy adults (20), might play a role in cognitive performance both in diabetic group and non-diabetic control. However, compared with controls, patients with diabetes had greater incremental rate of both MoCA score and RAVLT from baseline to follow-up visit. Therefore, the observed cognitive improvement in patients with type 2 diabetes was not exclusively attributed to practice effects.

Interestingly, patients with type 2 diabetes had higher MoCA than controls at the follow-up visit, indicating better global cognitive function in diabetic group. A possible explanation might be the neuroprotective effect of some antidiabetic agents (21) such as glucagon-like peptide-1 receptor agonist (22) and metformin (23), which were widely used in this study cohort at the follow-up visit. Recently, the REWIND trial (24), which also used MoCA as one of the two primary endpoints, revealed that glucagon-like peptide-1 receptor agonist might reduce cognitive impairment in patients with type 2 diabetes. Therefore, randomize controlled study is needed to investigate the relationship between the antidiabetic drugs, brain functional connectivity and cognitive outcome.

The mean HbA1c level moderated from 10% to 6.5% during the study period in patients with type 2 diabetes. However, no association was observed between the changes in the HbA1c level and the changes in brain functional connectivity. Moreover, no association between the changes in any other hyperglycemia-related variables (i.e., fasting plasma glucose level, fasting serum C-peptide level, HOMA-%β, and HOMA-IR) and the changes in brain connectivity was found. This result indicated that hyperglycemia-related variables in peripheral blood might not be sufficient to specifically reflect the brain activity. For example, the brain does not depend on insulin to use glucose, and the insulin action in the central nervous system is substantially different from that in peripheral tissues (25). Furthermore, some antidiabetic drugs might increase insulin sensitivity or improve glucose uptake in the brain (1). Therefore, in addition to lowering blood glucose, antidiabetic drugs might have a direct effect on brain functional connectivity. It further complicated the intricate relationship between hyperglycemia and brain functional connectivity. It is possible that no direct or specific link exists between peripheral hyperglycemia-related variables and brain function. Instead, fMRI signals are thought to be a valuable tool to unmask brain activity and are well related to cognitive changes. Therefore, in addition to concerns regarding early metabolic control in patients with type 2 diabetes, researchers should pay more attention to the changes in central nervous system (e.g., brain neuroimaging marker) in the future.

Regarding brain morphometry, no between-group differences (at both voxel and global levels) were observed at both the time points, supporting our previous hypothesis that functional changes in the brain occurred before structural changes (11). However, the stage at which substantial changes in brain structure occur during type 2 diabetes progression and effects of structural atrophy on cognitive dysfunction are unclear.

This study has several limitations. First, the current study was an observational study. Hence, whether cognitive reversal is related to a certain intervention is unclear. A well-designed randomized controlled study is required to elucidate whether the cognitive benefits are attributed to the “window period,” to certain interventions, or both. Second, it is still unclear whether the preclinical stage would be present in the middle-aged to elderly population. Finally, the conclusions from this pilot study should be used with caution because relatively small number of subjects were included. A study with longer follow-up and larger sample size is required to verify the current results.

In conclusion, the current study demonstrates the recovery of cognitive function coupled with the normalization of brain functional hyperconnectivity. These findings suggest that diabetes-related cognitive dysfunction is not a one-way process. There might be a potential “window period” for cognitive reversal in the early stage of type 2 diabetes, and the enhancement of brain functional connectivity may serve as the marker.

## Data Availability Statement

The raw data supporting the conclusions of this article will be made available by the authors, without undue reservation.

## Ethics Statement

The studies involving human participants were reviewed and approved by Shanghai General Hospital Ethics Committee. The patients/participants provided their written informed consent to participate in this study.

## Author Contributions

FF researched data and wrote the manuscript. Y-JG performed cognitive testing. QL and R-BG performed MRI scanning. MK contributed to the statistical analysis of clinical data. M-MM detected the diabetic retinopathy. DM collected the clinical data. D-ZY and LZ oversaw MRI analyses. Y-FW and D-ZY designed the study, reviewed and edited the manuscript. Y-FW and D-ZY are the guarantors of this work. All authors contributed to the article and approved the submitted version.

## Funding

This work was supported by the grants from National Natural Science Foundation of China (81900813 and 31600869), Science and Technology Commission of Shanghai Municipality (19411964500), Shanghai Hospital Development Center (2020CR4013), and Shanghai Jiao Tong University (ZH2018QNB10). D-ZY gratefully acknowledges the support from the Sanofi-Aventis Shanghai Institutes for Biological Sciences scholarship program.

## Conflict of Interest

The authors declare that the research was conducted in the absence of any commercial or financial relationships that could be construed as a potential conflict of interest.

## Publisher’s Note

All claims expressed in this article are solely those of the authors and do not necessarily represent those of their affiliated organizations, or those of the publisher, the editors and the reviewers. Any product that may be evaluated in this article, or claim that may be made by its manufacturer, is not guaranteed or endorsed by the publisher.
