# Supplementary figures and images for: Cognitive Dysfunction in Type 2 Diabetes Is Not a One-Way Process: Evidence From a Longitudinal Brain Connectivity Study
Source: Front Endocrinol (Lausanne). 2022 Apr 28;13:874538. doi: 10.3389/fendo.2022.874538 (PMC9095898; doi:10.3389/fendo.2022.874538)

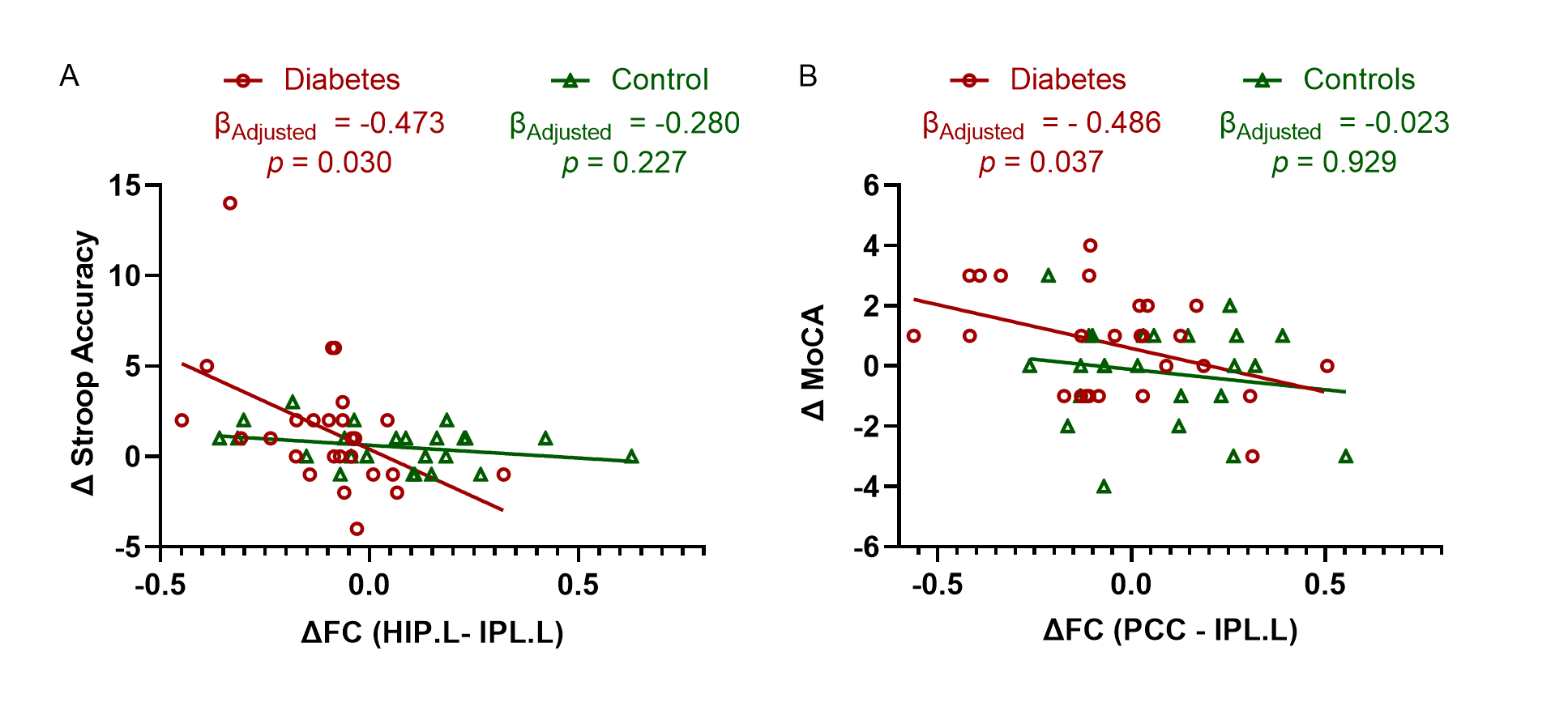

Supplement: Supplementary file 1 [file Image_1.tif]
