# Supplementary material for: Cognitive Dysfunction in Type 2 Diabetes Is Not a One-Way Process: Evidence From a Longitudinal Brain Connectivity Study
Source: Front Endocrinol (Lausanne). 2022 Apr 28;13:874538. doi: 10.3389/fendo.2022.874538 (PMC9095898; doi:10.3389/fendo.2022.874538)
Supplement: Supplementary file 2 [file Table_1.docx]

**Supplementary Table 1**. The baseline demographic and neuropsychological details of the withdrawn subjects.

|  | | **Controls** | | **Diabetes** | | |
| --- | --- | --- | --- | --- | --- | --- |
|  | **Withdrawn**  **N = 8** | **Followed**  **N =24** | ***P*** | **withdrawn**  **N = 9** | **Followed**  **N = 26** | ***P*** |
| **Age, y** | 36.5 (25.0,40.0) | 35.5 (25.0, 40.0) | 0.717 | 29.4 (23.0, 36.0) | 34.0 (24.0, 40.0) | 0.093 |
| **Male** | 6 (75.0) | 13 (54.2) | 0.271 | 5 (55.6) | 20 (76.9) | 0.211 |
| **Education, y** | 14.0 (11.0, 20.0) | 15.0 (6.0, 20.0) | 0.915 | 15.0 (8.0, 19.0) | 12.0 (8.0, 19.0) | 0.469 |
| **BMI, kg/m^2^** | 22.6 (19.8, 26.0) | 23.7 (18.2, 30.1) | 0.254 | 28.2 (22.7, 34.6) | 27.0 (18.4, 34.7) | 0.305 |
| **History of smoking** | 3 (37.5) | 10（41.7） | 0.587 | 3 (33.3) | 10（38.5） | 0.557 |
| **Presence of hypertension** | 4 (50.0) | 5（20.8） | 0.129 | 2 (22.2) | 8（30.8） | 0.488 |
| **Statin treatment** | 0 (0) | 0（0） | – | 2 (22.2) | 5（19.2） | 1.000 |
| **HbA1c (%)** | 5.5 (4.8, 5.7) | 5.6 (5.0, 6.1) | 0.566 | 12.4 (5.9, 14.3) | 10.5 (6.3, 13.3) | 0.110 |
| **Fasting plasma glucose (mmol/L)** | 4.8 (4.3, 5.6) | 4.8 (3.8, 5.9) | 0.685 | 9.0 (4.4, 20.0) | 7.1 (3.4, 16.9) | 0.897 |
| **Fasting serum C-peptide (pmol/L)** | 516.0 (328.0, 673.0) | 468.5 (266.0, 993.0) | 0.700 | 425.5 (226.0, 599.0) | 520.5 (239.0, 1224.0) | 0.516 |
| **HOMA-%β** | 100.8 (74.3, 150.3) | 102.5 (62.4, 210.5) | 0.976 | 52.3 (4.9, 149.4) | 49.9 (10.1, 114.0) | 0.985 |
| **HOMA-IR** | 1.10 (0.70, 1.50) | 1.01 (0.59, 2.09) | 0.700 | 1.26 (0.87, 1.82) | 1.32 (0.48, 4.10) | 0.753 |
| **Total Cholesterol (mmol/L)** | 5.22 (3.34, 6.22) | 4.75 (2.98, 7.40) | 0.328 | 5.07 (3.42, 6.12) | 4.96 (3.47, 6.80) | 0.516 |
| **Triglyceride (mmol/L)** | 1.09 (0.83, 2.77) | 1.11 (0.39, 2.89) | 0.901 | 1.05 (0.76, 1.56) | 2.58 (0.60, 6.29) | 0.210 |
| **HDL cholesterol (mmol/L)** | 1.42 (0.93, 1.57) | 1.22 (0.86, 2.09) | 0.566 | 1.01 (0.95, 1.10) | 0.86 (0.62, 1.50) | 0.446 |
| **LDL cholesterol (mmol/L)** | 3.32 (2.09, 4.38) | 3.18 (1.51, 5.02) | 0.381 | 3.57 (2.06, 3.90) | 3.14 (0.94, 4.39) | 0.670 |
| **Uric acid (μmol/L)** | 326 (179, 409) | 293 (185, 463) | 0.823 | 319 (209, 373) | 343 (175, 635) | 0.753 |
| **Urine ACR (mg/g)** | 11.0 (6.1, 15.1) | 9.38 (4.5, 21.7) | 0.354 | 20.5 (3.4, 29.4) | 14.0 (2.1, 29.9) | 0.810 |
| **Diabetes duration (year)** | – | – | – | 0.83 (0.1, 9.0) | 1.5 (0, 10) | 0.590 |
| **Cognitive Performance** |  |  |  |  |  |  |
| **MoCA** | 28.0 (25.0, 29.0) | 28.0 (21.0, 30.0) | 0.743 | 27.5 (27, 30) | 28.0 (22.0, 30.0) | 0.883 |
| **Stroop Accuracy** | 49.0 (49.0, 50.0) | 49.0 (47.0, 50.0) | 0.321 | 49.5 (47.0, 50.0) | 49.0 (36.0, 50.0) | 0.425 |
| **Stroop Reaction Time** | 55.0 (40.8, 55.2) | 58.2 (33.0, 90.0) | 0.191 | 51.2 (48.0, 60.0) | 67.3 (44.8, 86.1) | 0.009^*^ |
| **RAVLT** | 36.0 (23.0, 49.0) | 40.0 (22.0, 53.0) | 0.462 | 36.0 (30.0, 38.0) | 35.0 (23.0, 56.0) | 0.976 |
| **VFT** | 57.0 (47.0, 63.0) | 69.0 (31.0, 85.0) | 0.129 | 63.5 (60.0, 69.0) | 60.0 (44.0, 85.0) | 0.498 |
| **BNT** | 25.0 (20.0, 29.0) | 27.0 (16.0, 30.0) | 0.631 | 26.0 (24.0, 28.0) | 27.0 (19.0, 29.0) | 0.837 |

Data are represented as median (range) or n (%). ^*^*P* <0.05.

HOMA-%β, homeostasis model assessment of β-cell function; HOMA-IR, homeostasis model assessment of insulin resistance; HDL, high-density lipoprotein; LDL, low-density lipoprotein; ACR, albumin-to-creatinine ratio; MoCA, Montreal Cognitive Assessment; Stroop Accuracy, the accuracy of Stroop Color Word Test – part C; Stroop Reaction Time, the reaction time of Stroop Color Word Test – part C; RAVLT, Rey auditory verbal learning test; VFT, Verbal fluency test; and BNT, Boston naming test.

|  | Medicated  *N* = 11 | Non-Medicated  *N* = 15 | *P* |
| --- | --- | --- | --- |
| **Baseline Cognition** |  |  |  |
| MoCA | 28.0 (22.0, 30.0) | 29.0 (26.0, 30.0) | 0.164 |
| Stroop Accuracy | 48.0 (36.0, 50.0) | 49.0 (44.0, 50.0) | 0.259 |
| Stroup Reaction Time (s) | 66.0 (44.8, 82.0) | 68.5 (53.5, 86.0) | 0.721 |
| RAVLT | 35.0 (25.0, 56.0) | 35.0 (23.0, 50.0) | 0.760 |
| VFT | 62.0 (44.0, 80.0) | 58.0 (47.0,85.0) | 0.474 |
| BNT | 27.0 (19.0, 28.0) | 26.0 (19.0, 29.0) | 0.646 |
| **Baseline Fuctional Connectivity** |  |  |  |
| HIP.L-IFG.L | 0.267 (0.110, 0.380) | 0.278 (0.010,0.790) | 0.384 |
| HIP.L-IPL.L | 0.197 (-0.027, 0.473) | 0.294 (0.032, 0.747) | 0.281 |
| PCC-IPL.L | 0.382 (0.162, 0.769) | 0.475 (0.160, 0.746) | 0.032^*^ |
| **Changes in Cognition** |  |  |  |
| MoCA | 2.0 (-1.0, 4.0) | 1.0 (-3.0, 3.0) | 0.134 |
| Stroop Accuracy | 1.0 (-4.0, 14.0) | 0.0 (-2.0, 6.0) | 0.474 |
| Stroup Reaction Time (s) | -3.2 (-18.0, 14.9) | -5.7 (-26.0, 14.0) | 0.959 |
| RAVLT | 5.0 (-7.0, 22.0) | 3.0 (-5.0, 15.0) | 0.838 |
| VFT | -1.0 (-8.0, 14.0) | 2.0 (-19.0,14.0) | 0.760 |
| BNT | 0.0 (-1.0,4.0) | 0.0 (-1.0, 3.0) | 0.878 |
| **Changes in Functional Connectivity** |  |  |  |
| HIP.L-IFG.L | -0.178 (-0.552, 0.096) | -0.089 (-0.472, 0.149) | 0.259 |
| HIP.L-IPL.L | -0.085 (-0.449, -0.030) | -0.071 (-0.389, 0.321) | 0.217 |
| PCC-IPL.L | 0.023 (-0.449, -0.030) | -0.110 (-0.562, 0.505) | 0.330 |
| Data were presented in Median (Range) | | | |

**Supplementary Table 2**. Subgroup analyze in patients with antidiabetic drugs and those without antidiabetic drugs at baseline.

**Supplementary Table 3**. Global brain volumes at baseline and at the follow-up visit.

|  |  | **Baseline** | | | **Follow-up** | | |
| --- | --- | --- | --- | --- | --- | --- | --- |
|  | **Controls**  **N = 24** | | **Diabetes**  **N = 26** | ***P* values** | **Controls**  **N = 24** | **Diabetes**  **N = 26** | ***P* values** |
| **Grey matter volume (mL)** | 620.8 ± 47.2 | | 643.7 ± 58.2 | 0.138 | 624.8 ± 42.7 | 646.9 ± 61.1 | 0.150 |
| **White matter volume (mL)** | 539.7 ± 47.9 | | 551.3 ± 61.7 | 0.466 | 540.6 ± 48.5 | 554.9 ± 63.1 | 0.379 |
| **Ventricular CSF volume (mL)** | 225.5 ± 28.3 | | 237.3 ± 33.3 | 0.188 | 223.3 ± 29.5 | 235.3 ± 32.5 | 0.183 |
| **Total brain volume (mL)** | 1386.0 ± 103.4 | | 1432.4 ± 138.2 | 0.191 | 1388.7 ± 102.4 | 1437.1 ± 140.0 | 0.174 |

Data are represented as mean ± SD.

**Supplemental table 4**. Associations between the changes of functional connectivity and the changes of hyperglycemia-related variables in patients with type 2 diabetes.

| **Functional Connectivity** | **Clinical Parameter** | **β_Adjusted_** | ***P*** |
| --- | --- | --- | --- |
| Δ HIP.L – IFG.L | Δ HbA1c | 0.097 | 0.695 |
|  | Δ Fasting plasma glucose | 0.156 | 0.512 |
|  | Δ Fasting serum C-peptide | -0.343 | 0.143 |
|  | Δ HOMA-%β | -0.196 | 0.448 |
|  | ΔHOMA-IR | -0.197 | 0.395 |
| Δ HIP.L – IPL.L | Δ HbA1c | -0.027 | 0.912 |
|  | Δ Fasting plasma glucose | 0.085 | 0.716 |
|  | Δ Fasting serum C-peptide | -0.023 | 0.922 |
|  | Δ HOMA-%β | 0.049 | 0.848 |
|  | ΔHOMA-IR | 0.020 | 0.931 |
| Δ PCC – IPL.L | Δ HbA1c | 0.267 | 0.220 |
|  | Δ Fasting plasma glucose | 0.264 | 0.207 |
|  | Δ Fasting serum C-peptide | -0.057 | 0.791 |
|  | Δ HOMA-%β | 0.066 | 0.775 |
|  | ΔHOMA-IR | 0.066 | 0.751 |

Δ is the value obtained after subtracting the baseline value from the follow-up value. HIP.L-IFG.L, the connectivity of the left hippocampus with the left inferior frontal gyrus; HIP.L-IPL.L, the connectivity of the left hippocampus with the left inferior parietal lobule; PCC-IPL.L, the connectivity of the posterior cingulated cortex with the left inferior parietal lobule. β are adjusted for age, sex, education level and follow-up interval.
